# Tracking the evolution and geographic spread of Influenza A

**DOI:** 10.1371/currents.RRN1014

**Published:** 2009-09-24

**Authors:** Donovan Parks, Norman MacDonald, Robert Beiko

**Affiliations:** ^*^Faculty of Computer Science, Dalhousie University and ^†^Dalhousie University

## Abstract

The 2009 swine-origin strain of Influenza A H1N1 has spread to nearly all parts of the world, with 175 countries reporting confirmed cases thus far. Consistent with seasonal flu outbreaks, the current pandemic strain has shown rapid dispersal, with multiple examples of introduction into different geographic regions. Here we use an automated pipeline to collect data for analysis in the geospatial package GenGIS, which allows the geographic and temporal tracking of new sequence types and polymorphisms. Using this approach, we examine a pair of amino acid changes in the neuraminidase protein that are implicated in antibody recognition, and exhibit global dispersal with little or no geographic structure.

## Introduction

Current best estimates put the common ancestor of the current pandemic 2009 H1N1 strain of swine-origin Influenza A virus (S-OIV: also referred to as H1N1pdm) in the fourth quarter of 2008 or very early in 2009 [1] [2]. The first significant outbreak thought to be associated with this strain occurred in La Gloria, Veracruz, Mexico in February 2009. Recognition of the novelty and pandemic potential of this strain occurred within the following two months, and ever since this strain has been the subject of an unprecedented campaign of monitoring, sequencing and analysis.

Transmission and lethality rates in humans vary drastically among Influenza A subtypes and this novel strain, which arose from a 'pseudo-antigenic shift' [3] introducing a very divergent H1 allele from swine, is still in the early stages of characterization [4] [5]. Initial isolates lacked many of the hallmarks of antiviral resistance that are now widespread in seasonal flu strains, but recent sequencing and laboratory analysis has confirmed the emergence of novel H275Y mutations that confer resistance to oseltamivir [6]. Although the isolates from the current pandemic outbreak are genetically very similar, there are many novel amino acid changes which may confer as yet unknown functional differences to the virus.

Previous studies have aimed to characterize the transmission dynamics of Influenza A during seasonal and pandemic outbreaks. While widespread travel and high population density contribute to rapid global dispersal, it may yet be possible to track the transmission of variants that bear characteristic polymorphisms [7]. Highly informative geovisualizations of Influenza A and multicellular organisms have been performed using Google Earth and plugins to commercial GIS software [8] [9]. Here we demonstrate an automated pipeline for retrieving and parsing Influenza isolates that can expedite the search for important patterns within an outbreak by allowing the geographic and temporal structure of these isolates to be visualized using GenGIS [10]. GenGIS is open source, complementing the free availability of all cartographic, location, and sequence data used in this analysis. Since GenGIS is custom-built with genes and phylogenetic trees in mind, a wider range of visualization and analysis tools are available.

## Methods

### Sequence Acquisition and Attribution

All S-OIV sequences examined in this paper were downloaded from the Influenza Virus Resource at NCBI (http://www.ncbi.nlm.nih.gov/genomes/FLU/SwineFlu.html). We implemented a pipeline whereby sequence data and isolate metadata are automatically acquired and parsed to give a 'first pass' of the geographic and temporal distribution of sequences: for example, records with an EpiFlu annotation block will typically contain the date and location of collection. Isolates lacking such data are inferred from context (e.g., isolate name) wherever possible. Location names are converted to latitude / longitude coordinates by querying the GeoNames server (http://www.geonames.org/). A total of 1624 S-OIV segments from 203 fully sequenced isolates were downloaded on August 11, 2009. 

### Multiple Sequence Alignment and Phylogenetic Inference

Although most full-length sequences in the 2009 S-OIV dataset are of exactly equal length for a given segment and are therefore not expected to possess insertion or deletion mutations, we still perform multiple sequence alignment to ensure this, and to cover cases where partial sequences must still be accurately aligned with their full-length counterparts. Among the multiple sequence alignment programs we tried, we found that MUSCLE version 3.7 [11] with a gap opening penalty of 20 and a gap extension penalty of 0 was least likely to introduce inaccurate internal gaps into the alignment; all segments were consequently aligned using these settings. Alignments were performed on eight protein sequences (PB2, PB1, PA, HA, NP, NA, M1, NS1); each of the resulting alignments was then used as a scaffold to align the corresponding DNA sequences to ensure preservation of the correct reading frame. A concatenate of the eight alignments was then constructed, with proteins added in the order indicated above, which corresponds to decreasing sequence length.

The 203 aligned S-OIV sequences were subjected to phylogenetic inference using RAxML version 7.0.4 [12], using a four-category discrete approximation of gamma-distributed rates across sites, a general time reversible model of nucleotide substitution, and 100 fast bootstrap replicates. Internal nodes with bootstrap support < 70% were collapsed prior to geographic and temporal analysis. Due to the difficulty in selecting a suitable outgroup for complete S-OIV sequences, mid-point rooting as implemented in MEGA 4.1 [13] was used to root the tree after removing the atypically long branch leading to sequence A/Zhejiang/2/2009. In examining a concatenate, we are implicitly assuming that no reassortment has occurred involving non-identical parental genomes. Reassortment has been demonstrated previously in Influenza A [14] [15] [16], but our initial searches for reassortment in S-OIV were inconclusive (data not shown), largely owing to the relatively small number of sequence substitutions within this set.

### Geospatial and Temporal Analysis Using GenGIS

GenGIS version 1.05 was used to visualize sequence polymorphisms and phylogenetic relationships in the context of geography and time. A global map was assembled and formatted from the GTOPO30 data set (http://edc.usgs.gov/products/elevation/gtopo30/gtopo30.html) using the GDAL (http://www.gdal.org/) open source geospatial libraries: see the GenGIS wiki (http://kiwi.cs.dal.ca/GenGIS/GTOP030_Tutorial) for a detailed description of map construction.

The following files were loaded into GenGIS in the indicated order: 

1. A world map covering the sample locations of interest;
2. A location file containing the latitude and longitude of each geographic location, each of which could contain one or more isolates;
3. A sequence file with one row per isolate, containing metadata information including collection dates and polymorphic amino acid sites;
4. A **geographic tree model (.GTM) file**, containing a Newick-formatted tree inferred using RAxML as described above. Three trees were examined separately in GenGIS:
5.
  - Aphylogenetic tree of 203 complete S-OIV sequences inferred using RAxML as described above;
  - A polymorphic subtree of 136 complete S-OIV sequences containing polymorphisms at NA positions 106 and 248; this subtree was selected for further analyses as it contains the majority of isolates where NA position 106 has the isoleucine (I) character that is found in other variants of the NA1 protein, and all isolates (except one) where NA position 248 has changed from an asparagine (N) to an aspartic acid (D).
  - A projected 'dispersal tree' of 16 complete S-OIV sequences demonstrating the rapid, global spread of S-OIV.

 We developed a series of scripts (available for download at http://kiwi.cs.dal.ca/GenGIS/Datasets) to recapitulate the emergence of interesting polymorphisms arising in sequences collected between April 1 and July 9, 2009.

## Results

### Sequence Properties and Phylogenetic Trees

The 203 genomes we analyzed using our pipeline were collected from three continents and a total of 13 countries: given the depth of sampling and geographic distribution within the United States, we further split this country into eight geographic regions. The dataset is dominated by sequences from New York, which is the source of 101 out of the 203 available genomes. Other countries represented were Canada (2 genomes), China (16), Denmark (2), Dominican Republic (4), France (6), Italy (4), Japan (22), Mexico (7), Russia (6), South Korea (1), Thailand (1), and the United Kingdom (1). Within the United States, the regions represented were Central Plains (4 genomes), Great Lakes (3), Mid-Atlantic (2), Northeast (103, including the 101 sequences from New York), Pacific (7), Rocky Mountains (2), Southeast (2) and Southwest (8). 91 genomes were available from isolates collected in April, 86 from May, 26 from June and 1 from July. Some genomes that were identical at every nucleotide position were observed, but these identical copies were retained because of the potential to give distinct geographic or temporal information. In total, 168 distinct genomic sequences were present in the data set. With such similar sequences, phylogenetic trees are expected to be poorly resolved: indeed, of the 200 internal nodes in the whole-genome tree, only 15 were supported by 90 or greater bootstrap replicates, a total of 47 were supported by at least 70 bootstrap replicates, while an even larger number had bootstrap support less than or equal to 10.

Among the alignment columns that were polymorphic within the S-OIV dataset, we found two in NA with possible antigenic implications. NA106, identified by [5] as part of the antigenic region within the neuraminidase domain of NA, is isoleucine in many distinct lineages of non-S-OIV NA proteins, but has mutated to valine in many of the S-OIV isolates. Among all non-S-OIV NA1 proteins examined in the NCBI nr database, a valine character at this position was found only in the isolate A/Bremen/5/2005(H1N1). Based on the state of the outgroup sequences, we treat isoleucine as the ancestral state for this character, and valine as a novel mutation within the S-OIV population. NA248 (NA2 numbering: NA247 [4]) is found in a region that is contacted by antibodies, is ancestrally asparagine but has mutated to glutamine in the 2009 S-OIV population. Figure 1 shows the phylogenetic tree inferred from the concatenated protein sequences. Many groups of sequences exhibiting the I106V shift descend from the backbone of the tree, and apart from the state implied by the majority of non-S-OIV NA1 sequences, it is unclear whether the founder of this group of sequences had an I or a V at this position. A completely supported grouping of I106 sequences, and a large grouping of N248D mutants which also contains another two sequences (A/Shanghai/71T/2009 and A/Italy/127/2009) with N at position 248, also descend from the backbone of the tree. An additional single isolate (A/Italy/05/2009) with the N248D mutation also descends from this backbone. Based on a simple parsimony argument given the tree, the common ancestor of this large N248D group appears to have been an N248D mutant, with subsequent reversions to N248. Reassortment could also produce these branching patterns. The double mutant type (V106, D248) is not observed in any of the isolate genomes.

**Figure:**
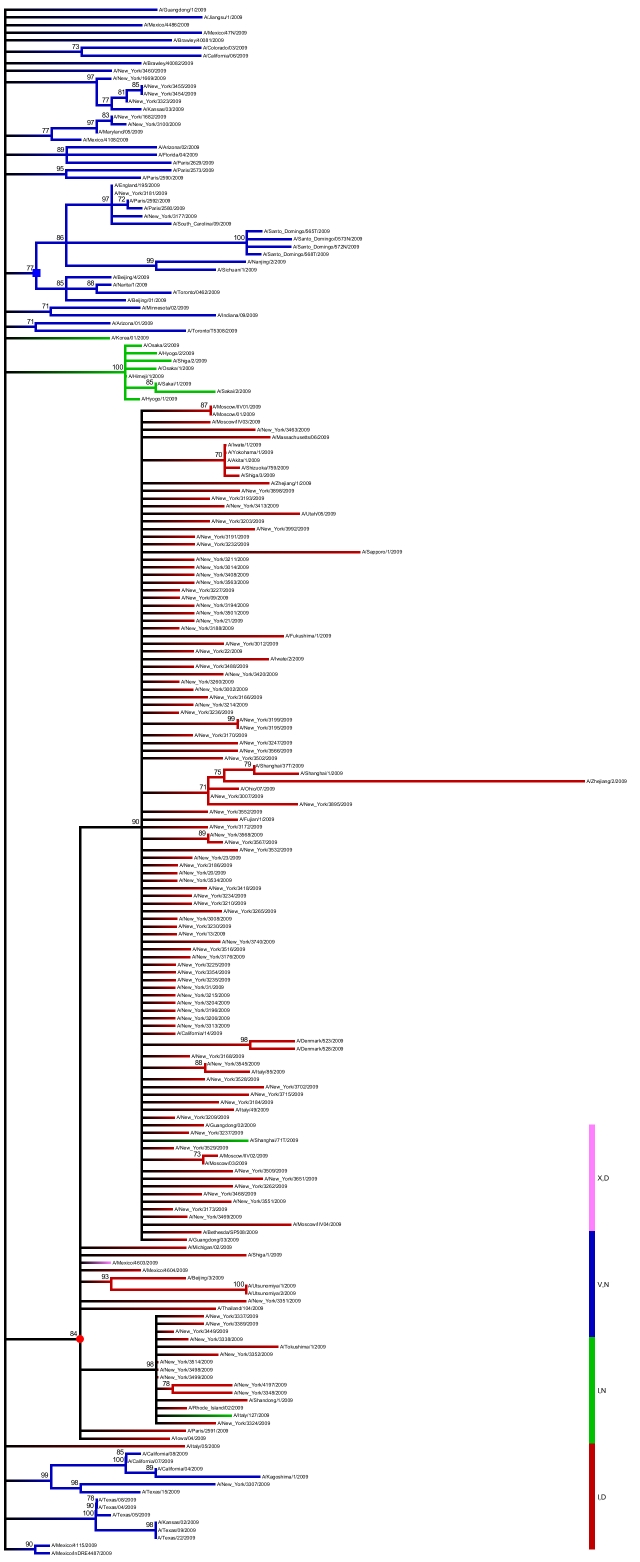
**Figure 1.** Phylogenetic tree of 203 S-OIV genomes, built using RAxML from a concatenated set of eight proteins. Displayed branch lengths are proportional to the observed number of substitutions per site. Only those internal nodes with bootstrap support >= 70% are shown as resolved, with the corresponding bootstrap proportion indicated on the tree. Branches are coloured based on the amino acid character states at NA positions 106 and 248; colour is assigned to a branch only if all its children have the same character state. The pink branch indicates uncertainty in the character at position NA106 in isolate A/Mexico/4603/2009. The "polymorphic" and "dispersal" trees selected for further analyses are denoted by a red circle and blue square, respectively. 

### Mapping Polymorphic Sites

The geographic context of the sequence polymorphisms can be gleaned from the isolate names in Figure 1, but a direct visualization gives a clearer picture of the geographic distribution of different polymorphic types. Figure 2 shows the distribution of polymorphisms at two different time points: (i) the set of genomes collected on or before April 27, which are entirely restricted to North America, and (ii) the complete set up to July 9, including several countries in Europe, Asia, and more sequences from North America. It is evident that this site was polymorphic in the population from an early stage. Neither the D state nor the N state is restricted to a particular geographic location: each of the three continents contains at least one location that is nearly uniformly D and one that is uniformly N. While this within-country homogeneity could very well be an artifact of low sampling effort, it serves to illustrate the rapid dispersal of mutant types. Movie 1 shows the entire time course of collected sequences, including the first observed sequence with D248, which was collected on April 20; the first sequenced genomes collected from Europe (England: April 28) and Asia (South Korea, May 2); and the first appearance of D248 in Europe (France: May 1) and Asia (Thailand: May 6). The rapid appearance of both sequence types across all locations suggests that both were established in the population prior to the sampling period.

**Figure:**
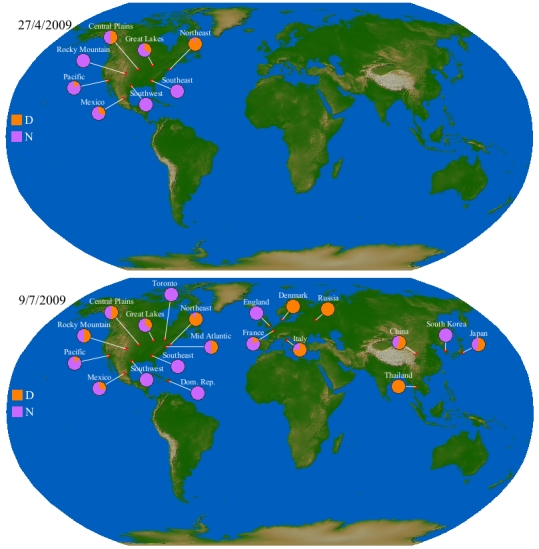
**Figure 2. **Relative proportion of isolates with asparagine (N) or aspartic acid (D) at NA position 248. Top and bottom images shows all isolates collected as of May 27 and July 9, respectively. Both amino acids are found in North America, Europe, and Asia.  

In Figure 3 we show the co-incidence of different character states at positions 106 and 248 of the NA protein. While the polymorphic types at both positions are cosmopolitan, it is interesting that the putatively ancestral I106 + N248 combination is observed in every Asian country save Thailand, which is represented by a lone sequence. The only other location where this combination was observed is Italy. The sequences from these four countries constitute at least three separate groupings in the tree: A/Shanghai/71T/2009 and A/Italy/127/2009 are the lone representatives from China and Italy respectively, while the single Korean genome and the cluster of sequences from Japan are sisters in a large multifurcating node in the tree. In spite of the massive sequencing effort concentrated in the northeastern United States, the observed sequences are almost all of the I106 + D248 type.

**Figure:**
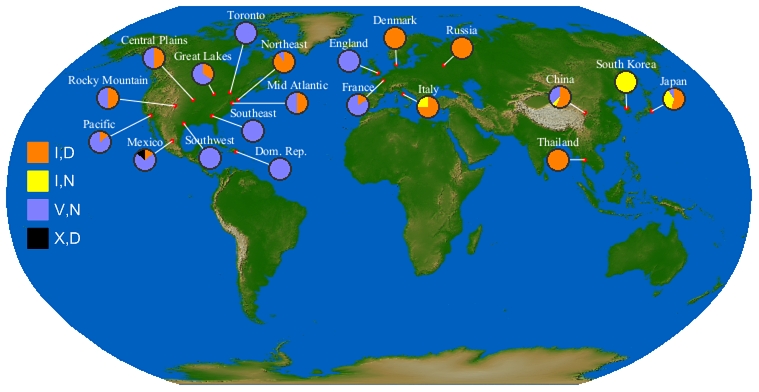
**Figure 3.** Relative proportion of isolates with isoleucine (I) or valine (V) at NA position 106, and asparagine (N) or aspartic acid (D) at NA position 248. 

### Geophylogenies

We mapped each tree leaf to its corresponding geographic location, and overlaid the tree in three dimensions (Figure 4). Although the tree can be viewed from any angle, zoom, and level of inclination in GenGIS, it is nonetheless difficult to discern the extent to which different groups of sequences cluster according to region, country or continent. White edges indicate ancestral branches whose children are found in more than one continent, so it is at least possible to reject the complete separation of isolates by continent. The two-dimensional tree view, in which the leaves of the tree are mapped to a geographic axis (Figure 5), more clearly shows the intermingling of isolates from different regions within the tree. While there appears to be some amount of geographic clustering based on inspection of the correlation lines that link tree leaves with geographic locations, such patterns must be interpreted with extreme caution when trees are highly multifurcating. When a single node has many children of equal rank, these can be arranged in any order to maximize the fit with geography. Closer inspection of the internal nodes of this tree reveal that many of these nodes subtend children of equal rank from all three continents. The branch-and-bound layout optimization and statistical test of significance with respect to geography [17] cannot currently be run on large trees with many multifurcating nodes; we require a projected subtree to rigourously test the relationship between phylogeny and geography.

**Figure:**
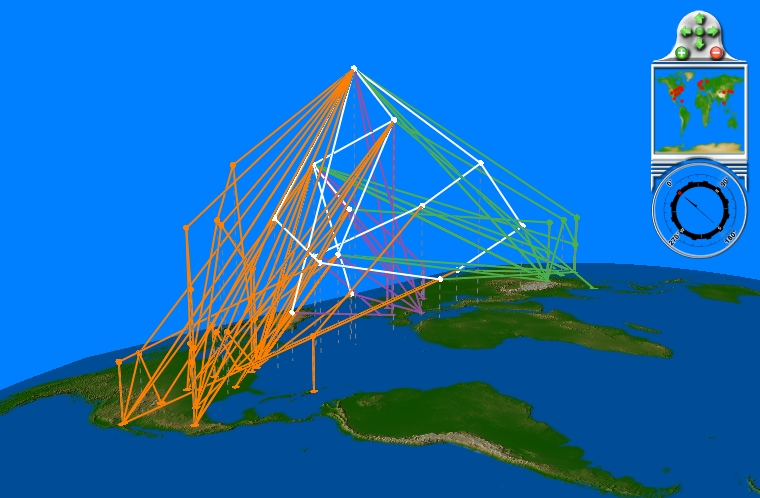
**Figure 4.** Geophylogeny of 203 S-OIV isolates with internal nodes containing less than 70% bootstrap support collapsed. Children of orange branches are contained within North America, purple within Europe, and green within Asia. White branches contain children from multiple continents and their presence in the tree indicates that isolates are not completely separated by continent.

**Figure:**
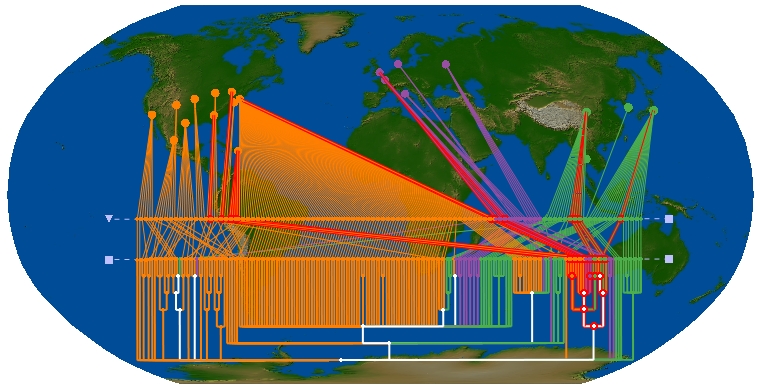
**Figure 5.** Two-dimensional tree displayed on a Robinson projection of the Earth. Branch colours are the same as specified in Figure 4. This visualization indicates that phylogenetically similar sequences are typically found in multiple continents. For example, the subtree highlighted in red contains isolates from North America, Europe, and Asia. 

To carry out a more effective test of the geographic relationships among S-OIV sequences, we constructed a projected "dispersal" tree that captures the strongly supported (boostrap >= 70%) relationships in the larger tree, but contains far fewer modifications and is therefore tractable for layout optimization and permutation test analysis. From Figure 6 it can be seen that geographic locations do not map to a common ancestor that excludes other locations; the North American and Asian sequences in particular are intermingled. We chose an east-west axis to lay out a two-dimensional version of the same tree (Figure 7), and used this axis as the basis for a permutation test. The null hypothesis of the permutation test is that there is no geographic structuring of the population along the proposed axis; rejection of this null hypothesis requires that a large majority of randomly permuted replicates show a greater number of crossings (i.e., a worse fit to the geographic axis) than does the original tree. Of the 1000 random reassignments of leaf nodes to geographic locations, 295 had equal or fewer crossings of location lines than did the original tree (p = 0.295), so we cannot reject the null hypothesis.

**Figure:**
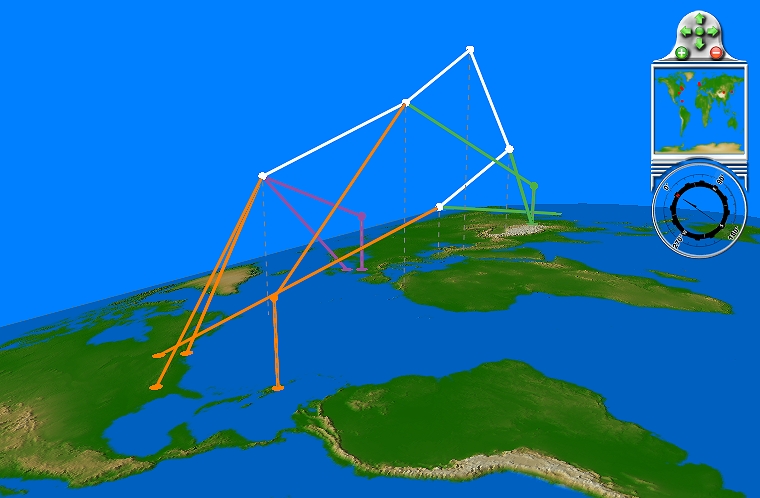
**Figure 6.** Geophylogeny of the 16-sequence "dispersal" subtree highlighted in Figure 5. All internal nodes are supported by at least 70 bootstrap replicates. Sequences within this subtree were collected in North America, Europe, and Asia.

**Figure:**
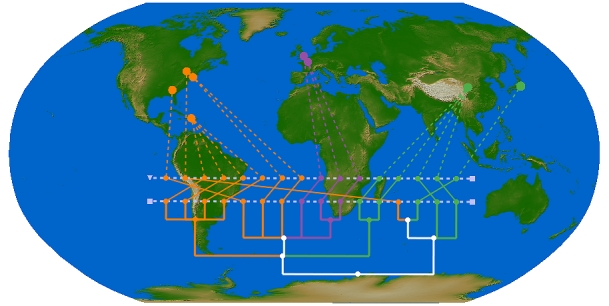
**Figure 7. **The "dispersal" subtree laid out along an east-west geographic axis. This axis also served as the reference for the permutation test described in the main text. 

Movie 2 shows the accumulation of sequences in the 'polymorphism subtree' which covers the large group of N248D mutants in our data set, starting from the first collected genome from Mexico on April 21. New sequences accumulate throughout this subtree for the entire duration of sampling, again in many cases likely corresponding to mutants that emerged prior to the beginning of the sampling phase. The left-hand side of the tree, which covers sequences from Mexico and the western half of the United States, shows a compelling example of distinct sequences that were already present in mid-April.

## Discussion

Previous pandemic and seasonal strains of Influenza A disseminated very rapidly throughout the world [18] [19], and given the outcomes of our visualizations and statistical test, this is clearly also the case for the recently emerged pandemic strain of H1N1. We have used phylogenetic analysis and GenGIS to highlight the distribution of both ancestral and derived amino acids for a pair of sites in the neuraminidase protein. The complete absence of the implied ancestral (I106, N248) type from North America is interesting, particularly in light of the tendency for most sequence variants to be represented in New York, which is both a global transit hub and the source of half the genomes in our data set. The appearance of putative revertants of this type in country-specific lineages is also intriguing: a V to I mutation is conservative, but the near-complete conservation of the I residue in other strains suggests that changes at this position are selectively unfavourable. Contrasting alternative hypotheses of reassortment, reversion, and a single point of origin (which would imply the recovery of an incorrect tree) is difficult given the lack of divergence among these sequences. Although the current pandemic is the most intensively sampled in history, it is clear that not all circulating sequence types have been sampled. Ideally the geophylogeny data would be complemented with information about the travel histories of affected individuals, but such combinations of clinical with molecular data raise significant privacy concerns. This is particularly true in cases where individuals have complicating conditions [6], or exhibit viral types with different transmission or drug resistance profiles.

Given the three-dimensional structure of the NA protein [5] [20], it appears unlikely that the two sites we examine above are functionally linked: they are localized to different regions within the protein and show no evidence of mutual interactions or interactions with a common ligand. Nonetheless, the association between these two polymorphic sites would produce a high mutual information value, given the surprising rarity of the putatively ancestral combination and the complete absence of a double mutant type. Mapping and interpreting these polymorphisms in light of a phylogenetic tree shows that their association could have very likely arisen by chance.

The relative rarity of sequence changes in this dataset led to our decision to use a concatenated whole-genome phylogeny to maximize the amount of resolution. As more mutations accumulate in the pandemic H1N1 strain, we will likely begin to see some of the characteristic hallmarks of Influenza A evolution and dispersal, including reassortment and the massive population bottlenecks that lead to very small effective population sizes. Characteristic mutations seen in other strains, such as the H275Y mutation that confers resistance to oseltamivir, are beginning to appear [6]; other adaptive mutations will likely be specific to this novel strain and will need to be interpreted in light of new functional assays. By combining sequence analysis with the visualization and analytical tools of GenGIS, we will be able to better understand the population dynamics of S-OIV as it continues to evolve and interact with other strains of influenza.

## Funding Sources

DHP is supported by the Killam Trusts. NJM is supported by an NSERC postgraduate scholarship. RGB acknowledges the support of the Canada Research Chairs program. The development of GenGIS was supported by Genome Atlantic, NSERC, and the Canada Foundation for Innovation.

## Competing Interests

The authors declare that they have no competing financial interests.
